# Supplementary material for: Protein phosphatase PP1 regulation of RNA polymerase II transcription termination and allelic exclusion of VSG genes in trypanosomes
Source: Nucleic Acids Res. 2024 May 23;52(12):6866–85. doi: 10.1093/nar/gkae392 (PMC11229358; doi:10.1093/nar/gkae392)
Supplement: gkae392_Supplemental_Files [file gkae392_supplemental_files.zip › Supplemental Figures Plus Legends 032724.pdf]

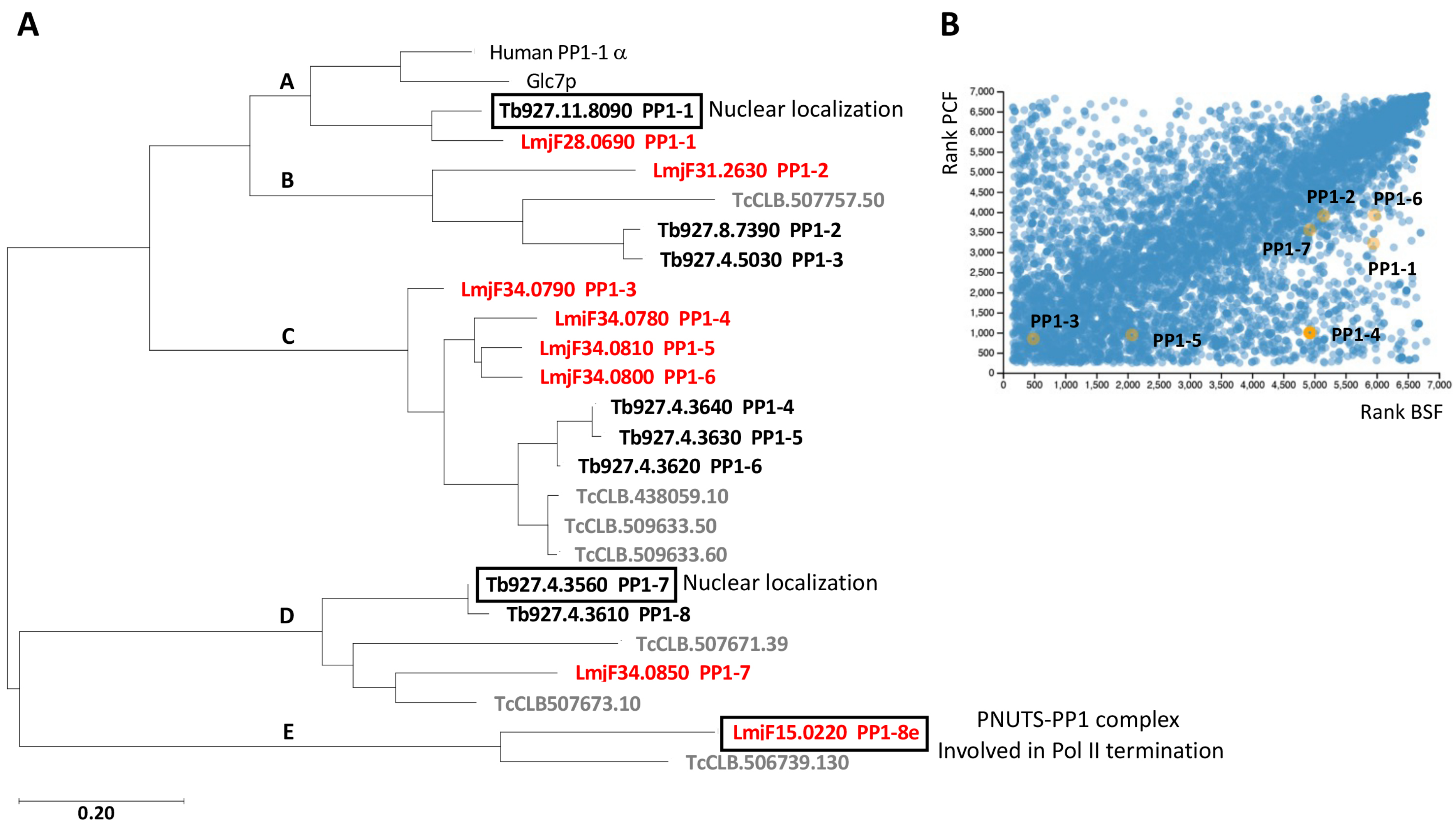
A
Human PP1-1 α
Glc7p
Tb927.11.8090 PP1-1
Nuclear localization
LmjF28.0690 PP1-1
LmjF31.2630 PP1-2
TcCLB.507757.50
Tb927.8.7390 PP1-2
Tb927.4.5030 PP1-3
LmjF34.0790 PP1-3
LmjF34.0780 PP1-4
LmjF34.0810 PP1-5
LmjF34.0800 PP1-6
Tb927.4.3640 PP1-4
Tb927.4.3630 PP1-5
Tb927.4.3620 PP1-6
TcCLB.438059.10
TcCLB.509633.50
TcCLB.509633.60
Tb927.4.3560 PP1-7
Nuclear localization
Tb927.4.3610 PP1-8
TcCLB.507671.39
LmjF34.0850 PP1-7
TcCLB507673.10
LmjF15.0220 PP1-8e
TcCLB.506739.130
PNUTS-PP1 complex
Involved in Pol II termination
0.20
B
Rank PCF
Rank BSF
PP1-1
PP1-2
PP1-3
PP1-4
PP1-5
PP1-6
PP1-7

**Figure S1**

```
PP1-1   0   MSVDVDAIIDKLLEV------------------------------------------RLS   18
PP1-2   0   MNCREIIRKLLLNPAHNNAATRTAQGDNGDSNQRAYTRISRLAAFQSAQTQESTPKTNGT   60
PP1-3   0   MNCDEIIKKLLLNPVHNTAATRGPAGENCESNQRTYTRISRLAAFQSAQTQESTPKTNGT   60
PP1-4   0   MTLVQTLLERMLSA-------------------------------------------KGN   17
PP1-5   0   MTLVQTLLERMLSA-------------------------------------------KGN   17
PP1-6   0   MTLVQTLLERMLSA-------------------------------------------KGS   17
PP1-7   31  --NRDRETIQRLVESILK-----------------------------------------D   47
PP1-8   0   ------------------------------------------------------------   0
human   8   --NLDSIIGRLLE-------------------------------------------VQGS   22
               A'

PP1-1       KPGKQVSLSENDVKNLVMRSREILLSQPALLELEAPIKICGDIHGQYYDLIRLFDNGGFP   78
PP1-2       GRATTEGLTEAEVRWLVMESRALFMSQPMLVEIAAPVRICGDVHGQYTDLLRLFDLGGFP   120
PP1-3       GRATTEGLTEAEVRWLVMESRALFMSQPMLVEIAAPVRICGDVHGQYTDLLRLFDLGGFP   120
PP1-4       TAQRQILIREEDIRTILNAVRDVFMSQPVVLDITPPVRICGDIHGQYYDLLRVFEKCGFP   77
PP1-5       TAQRQILIREEDIRTILNAVRDVFMSQPVVLDITPPVRICGDIHGQYYDLLRVFEKCGFP   77
PP1-6       ATQQQILIREEDIRTILNTVRDVFMSQPMLLEITPPVRICGDIHGQYYDLLRVFEKCGFP   77
PP1-7       WQSAQELLDGQIISTILRLVRPILMAQPMLVRLQAPINVCGDIHGQIGDLVEIFRAGGMP   107
PP1-8       ------------------------MAQPMLVRLQAPINVCGDIHGQIGDLVEIFRAGGMP   36
human       RPGKNVQLTENEIRGLCLKSREIFLSQPILLELEAPLKICGDIHGQYYDLLRLFEYGGFP   82
                                    ::** :: :  *:.:***:***  **:.:*   *:*
                           A              1      2           B

PP1-1       PSANYLFLGDYVDRGKQGLETICLVLAFKVKFPENFFILRGNHECASINRIYGFFDECKR   138
PP1-2       PDANYIFLGDYVDRGDQSLETICLLLAYKLSFPETFFLLRGNHECSSINRIYGFFDECKR   180
PP1-3       PDANYIFLGDYVDRGDQSLETICLLLAYKLSFPETFFLLRGNHECSSINRIYGFFDECKR   180
PP1-4       PYSNYLFLGDYVDRGRHSVETITLLFCYKIVYPENFFLLRGNHECASINKMYGFFDDVKR   137
PP1-5       PYSNYLFLGDYVDRGRHSVETITLLFCYKIVYPENFFLLRGNHECASINKMYGFFDDVKR   137
PP1-6       PYSNYLFLGDYVDRGRHSVETITLLFCYKIVYPENFFLLRGNHECASINKMYGFFDDVKR   137
PP1-7       PATNYLFLGDYVDRGKHGTEVIVVLLGLKILYPDGMCVLRGNHESDSICRIYGFFDEVKR   167
PP1-8       PATNYLFLGDYVDRGKHGTEVIVVLLGLKILYPDGMCVLRGNHESDSICRIYGFFDEVKR   96
human       PESNYLFLGDYVDRGKQSLETICLLLAYKIKYPENFFLLRGNHECASINRIYGFYDECKR   142
            * :**:********* :. *.* :::  *: :*: : :******. ** ::***:*: **
               3                 C             4        D      E

PP1-1       RYNIRLWKVFTDTFNCLPVACIIDDKIFCCHGGLSPDLQSMEQIKKIERPCDVADTGLIC   198
PP1-2       RYSVRLWKQFTDTFNCMPVAGLVEGRILCMHGGLSPELTDLDQIRRILRPTDVPDSGLIC   240
PP1-3       RYSVRLWKQFTDTFNCMPVAGLVEGRILCMHGGLSPELTDLDQIRRILRPTDVPDSGLIC   240
PP1-4       RYNIKLFKAFTDVFNTMPVCCVVGEKIICMHGGLSPDMTSLAAVNEIERPLDVPDKGILC   197
PP1-5       RYNAKLFKAFTDVFNTMPVCCVVGEKIICMHGGLSPDMTSLACVSEIERPLDVPDKGILC   197
PP1-6       RYNIKLFKAFTDVFNTMPVCCVVSEKIICMHGGLSPDMTSLASVNEIERPLDVPDKGILC   197
PP1-7       RFSVKLFKEFTDVFNCLPIAALIDDIALCMHGGLSPDLRNLRQIERIERPLIVPDTGLAC   227
PP1-8       RFSVKLFKEFTDVFNCLPIAALIDDIALCMHGGLSPDLRNLRQIERIERPLIVPDTGLAC   156
human       RYNIKLWKTFTDCFNCLPIAAIVDEKIFCCHGGLSPDLQSMEQIRRIMRPTDVPDQGLLC   202
            *:. :*:* *** ** :*:. ::    :* ******:: .:  : .* **  * * *: *
                 F              5      6               G             H

PP1-1       DLLWSDPEEGL-SGWGENDRGVSYTFGQDIVAKFLSRHDFDLIVRAHQVVEDGYQFFATR   257
PP1-2       DLLWSDPSTNMESNWSENDRGVSWTFSESVVKSFNKKFDLDLICRAHQVVDAGYEFFAAR   300
PP1-3       DLLWSDPSTNMESNWSENDRGVSWTFSESVVKSFNKKFDLDLICRAHQVVDAGYEFFAAR   300
PP1-4       DLLWADPEEEV-KGFLESDRGVSYLFGEDIVTDFLDMVDMDLVVRAHQVMERGYGFFANR   256
PP1-5       DLLWADPEEEV-KGFLESDRGVSYLFGEDIVTDFLDMVDMDLVVRAHQVMERGYGFFANR   256
PP1-6       DLLWADPEDEV-KGFLESDRGVSYLFGEDIVTDFLDMVDMDLVVRAHQVMERGYGFFANR   256
PP1-7       DLLWADPEEAG-SGWMESERGVSYTFGEDIVKETCERLDIDVVLRAHQVVDKGYQFFAGR   286
PP1-8       DLLWADPEEAG-SGWMESERGVSYTFGEDIVKETCERLDIDVVLRARF------QYFGSC   209
human       DLLWSDPDKDV-QGWGENDRGVSFTFGAEVVAKFLHKHDLDLICRAHQVVEDGYEFFAKR   261
            ****:**.    ..: *.:****: *. .:* .     *:*:: **:        :*.
                 7        8        9          I          10          11

PP1-1       QLITIFSAPNYCNEFDNSGAVMSVDADLLCSFQILKPSVKKPKYFQ--------------   303
PP1-2       QLVTVFSAPNYCDEFDNAGAFMCVDENLMCSFVQIEPTRTLLRYFF--------------   346
PP1-3       QLVTVFSAPNYCDEFDNAGAFMCVDENFMCSFIRIEPTRTLLRYFF--------------   346
PP1-4       QLVTIFSAPNYCGEFDNDAAVMNVDEKLQCSFAIIAAR----------------------   294
PP1-5       QLVTIFSAPNYCGEFDNDAAVMNVDEKLQCSFAIIAAR----------------------   294
PP1-6       QLVTIFSAPNYCGEFDNDAAVMNVDEKLQCSFLIIPAR----------------------   294
PP1-7       QLVTIFSASNYCGEFTNSGAMMIMDENCKCRFEIFRPQYD--------------------   326
PP1-8       ------------------------------------------------------------   209
human       QLVTLFSAPNYCGEFDNAGAMMSVDETLMCSFQILKPADKNKGKYGQFSGLNPGGRPITP   321
              12                  13        14
```

**Figure S2**

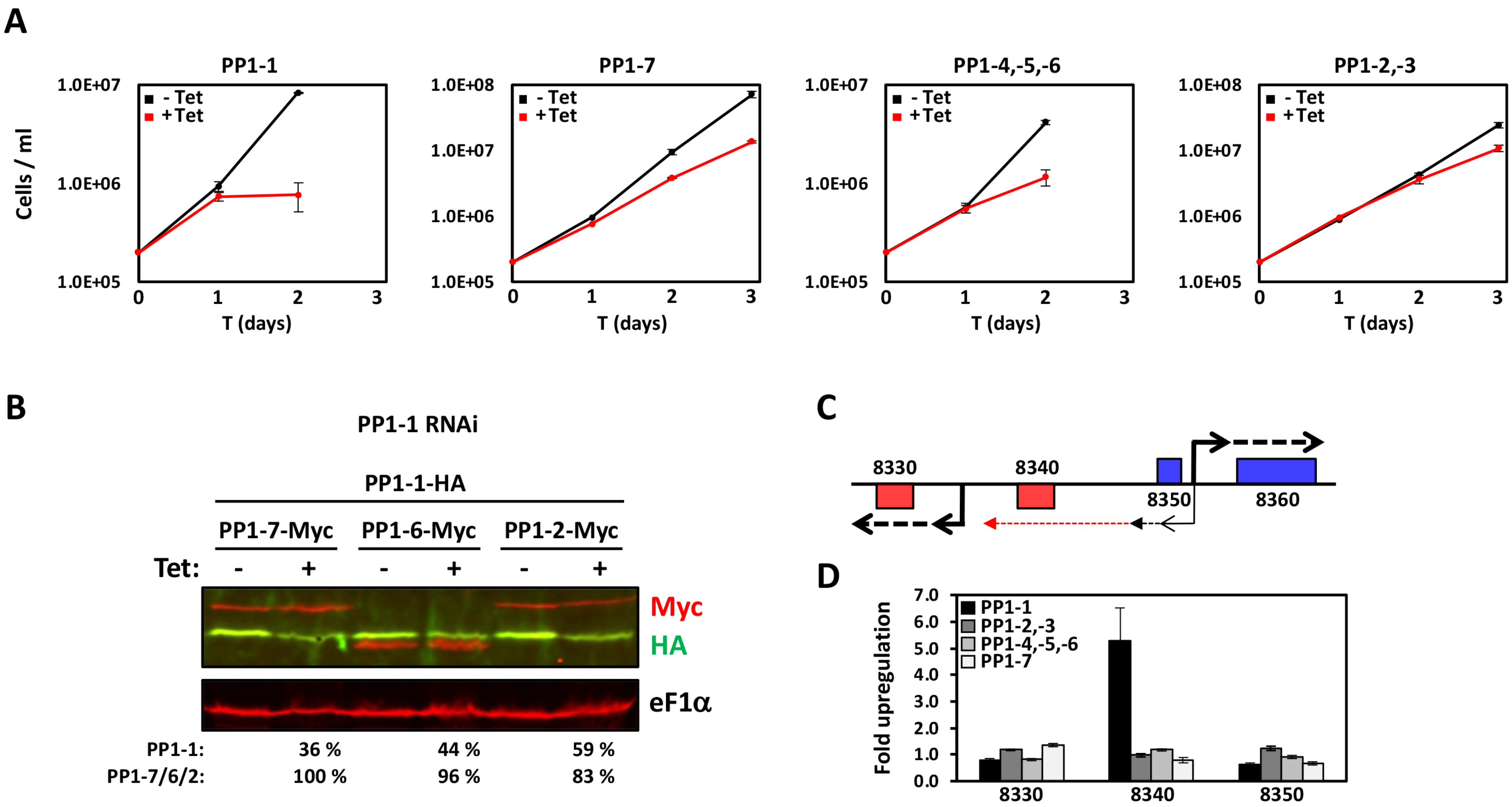
A
PP1-1
PP1-7
PP1-4,-5,-6
PP1-2,-3
Cells / ml
1.0E+08
1.0E+07
1.0E+06
1.0E+05
- Tet
+Tet
T (days)
0
1
2
3
B
PP1-1 RNAi
PP1-1-HA
PP1-7-Myc
PP1-6-Myc
PP1-2-Myc
Tet:
-
+
Myc
HA
eF1α
PP1-1: 36 % 44 % 59 %
PP1-7/6/2: 100 % 96 % 83 %
C
8330
8340
8350
8360
D
Fold upregulation
7.0
6.0
5.0
4.0
3.0
2.0
1.0
0.0
PP1-1
PP1-2,-3
PP1-4,-5,-6
PP1-7
8330
8340
8350

Figure S3

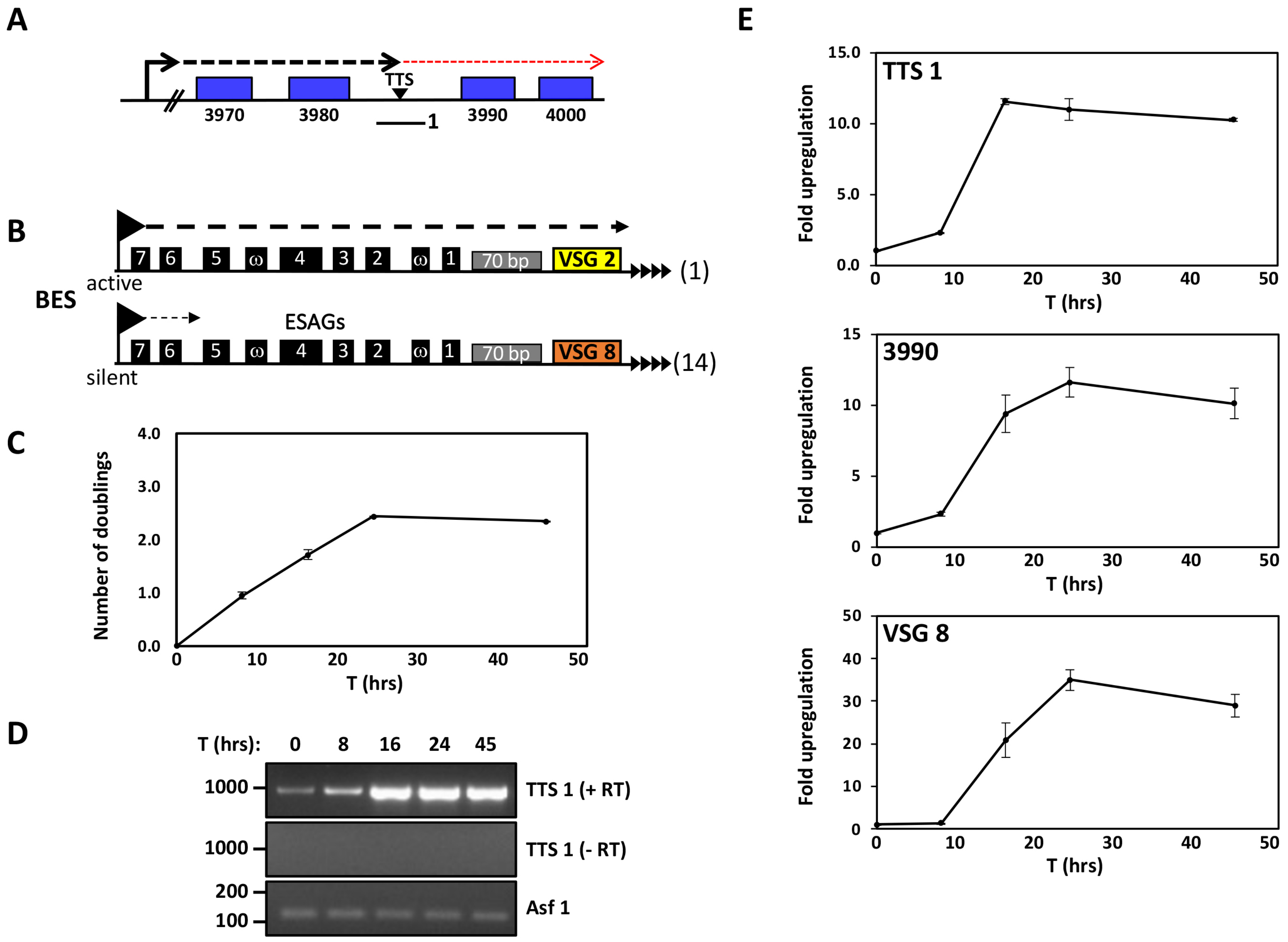
A
TTS
3970
3980
1
3990
4000
B
BES
active
silent
7
6
5
ω
4
3
2
ω
1
70 bp
VSG 2
(1)
ESAGs
VSG 8
(14)
C
Number of doublings
T (hrs)
D
T (hrs): 0 8 16 24 45
1000
TTS 1 (+ RT)
1000
TTS 1 (- RT)
200
100
Asf 1
E
TTS 1
3990
VSG 8
Fold upregulation
T (hrs)

Figure S4

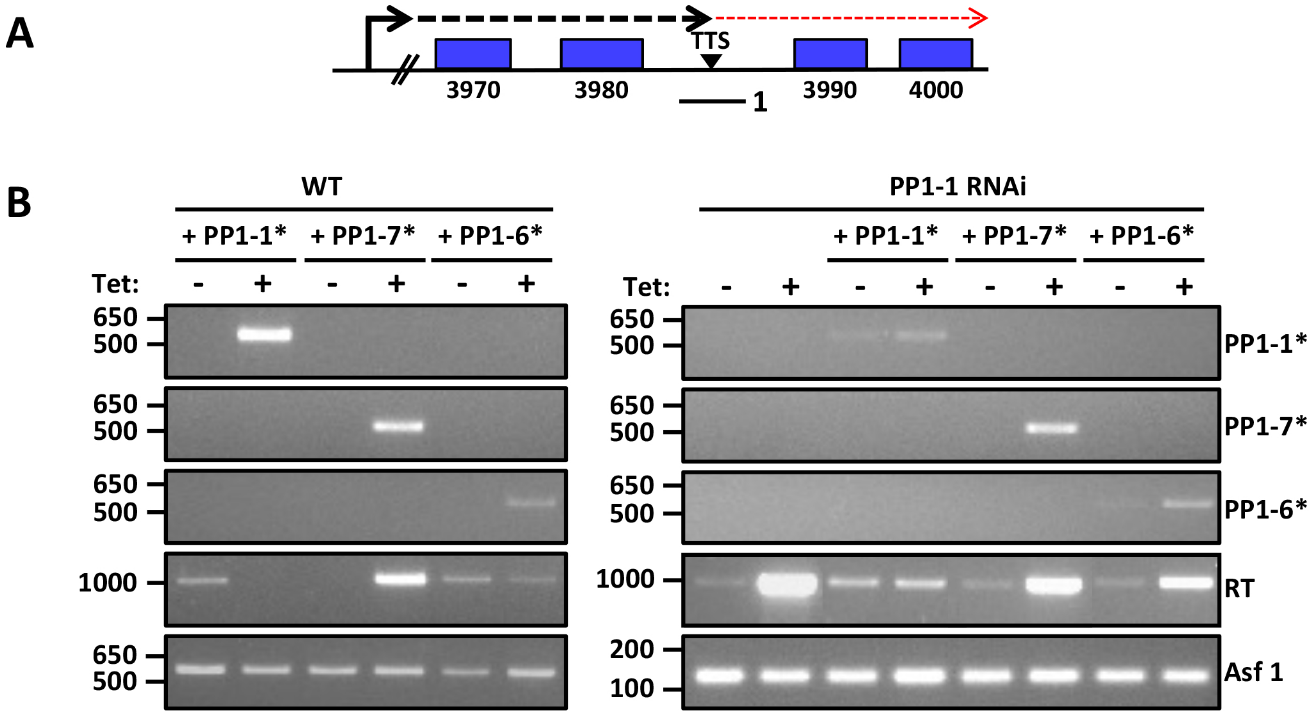
A
TTS
3970
3980
1
3990
4000
B
WT
+ PP1-1*
+ PP1-7*
+ PP1-6*
Tet:
-
+
-
+
-
+
650
500
1000
PP1-1 RNAi
+ PP1-1*
+ PP1-7*
+ PP1-6*
Tet:
-
+
-
+
-
+
-
+
PP1-1*
PP1-7*
PP1-6*
RT
200
100
Asf 1

Figure S5

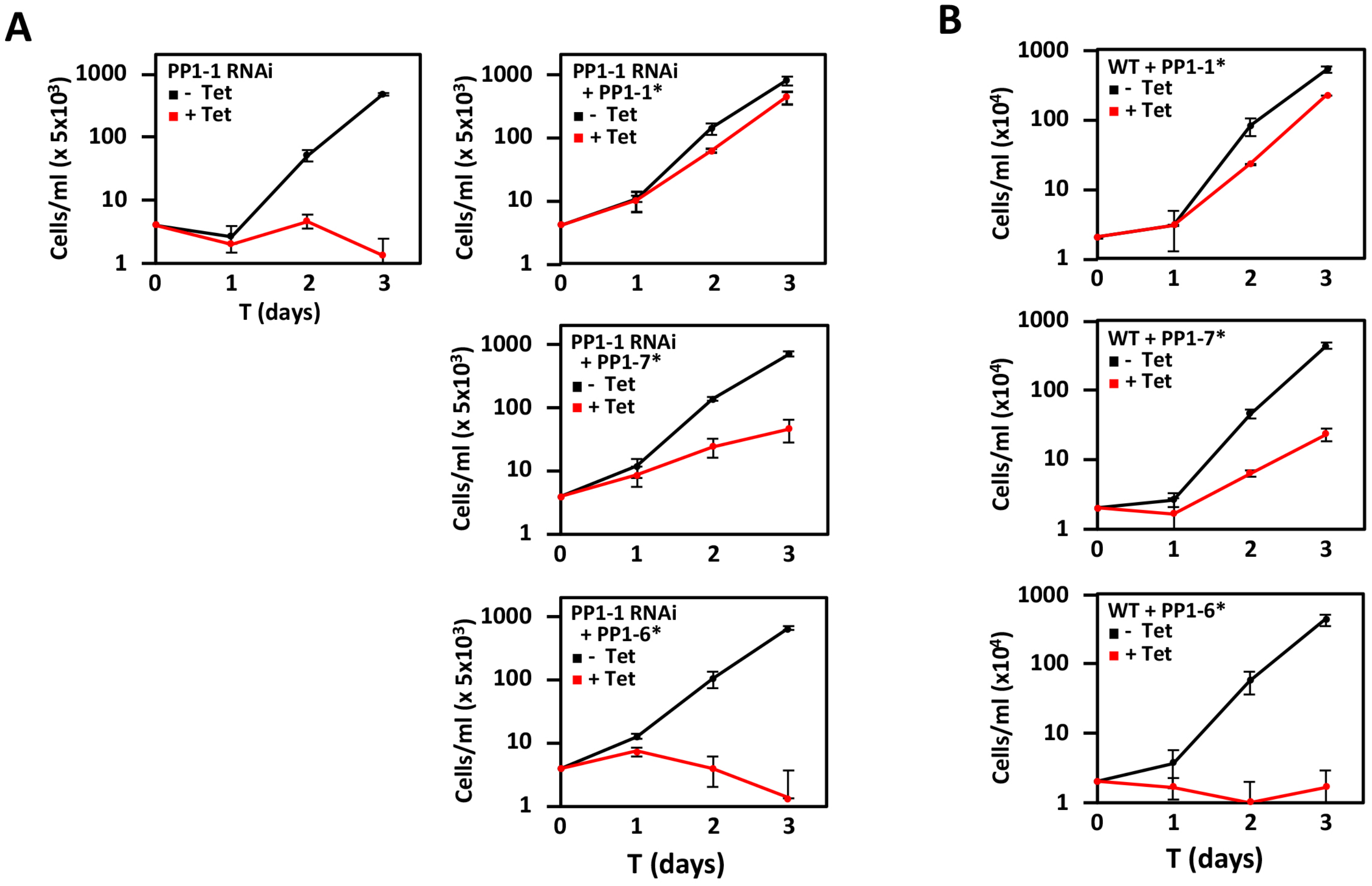
A
PP1-1 RNAi
- Tet
+ Tet
Cells/ml (x 5x10$^3$)
T (days)
PP1-1 RNAi + PP1-1*
PP1-1 RNAi + PP1-7*
PP1-1 RNAi + PP1-6*
B
WT + PP1-1*
WT + PP1-7*
WT + PP1-6*
Cells/ml (x10$^4$)

Figure S6

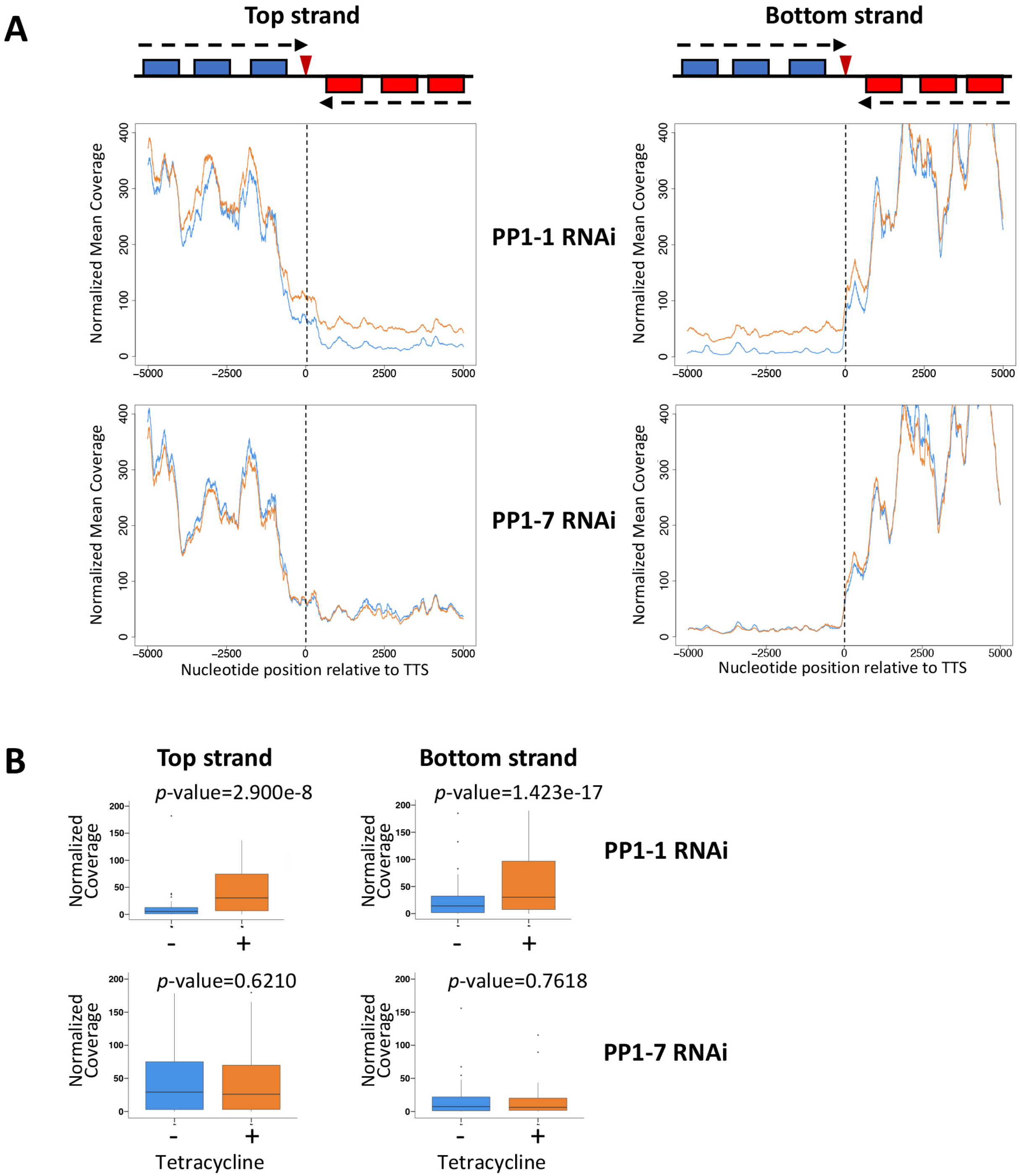
A
Top strand
Bottom strand
PP1-1 RNAi
PP1-7 RNAi
Normalized Mean Coverage
Nucleotide position relative to TTS
B
Top strand
Bottom strand
p-value=2.900e-8
p-value=1.423e-17
p-value=0.6210
p-value=0.7618
Normalized Coverage
Tetracycline
PP1-1 RNAi
PP1-7 RNAi

**Figure S7**

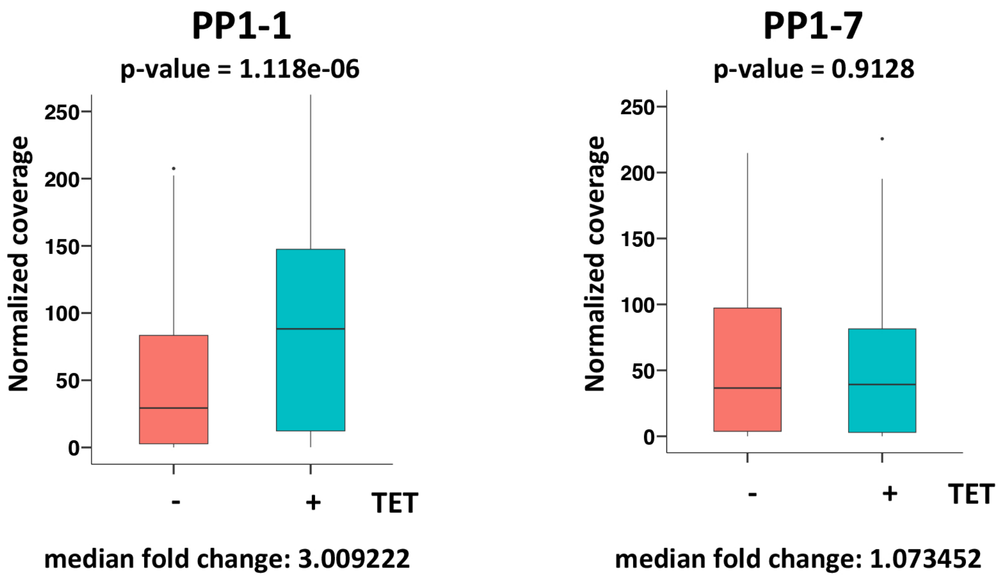
PP1-1
p-value = 1.118e-06
Normalized coverage
250
200
150
100
50
0
-
+
TET
median fold change: 3.009222
PP1-7
p-value = 0.9128
Normalized coverage
250
200
150
100
50
0
-
+
TET
median fold change: 1.073452

Figure S8

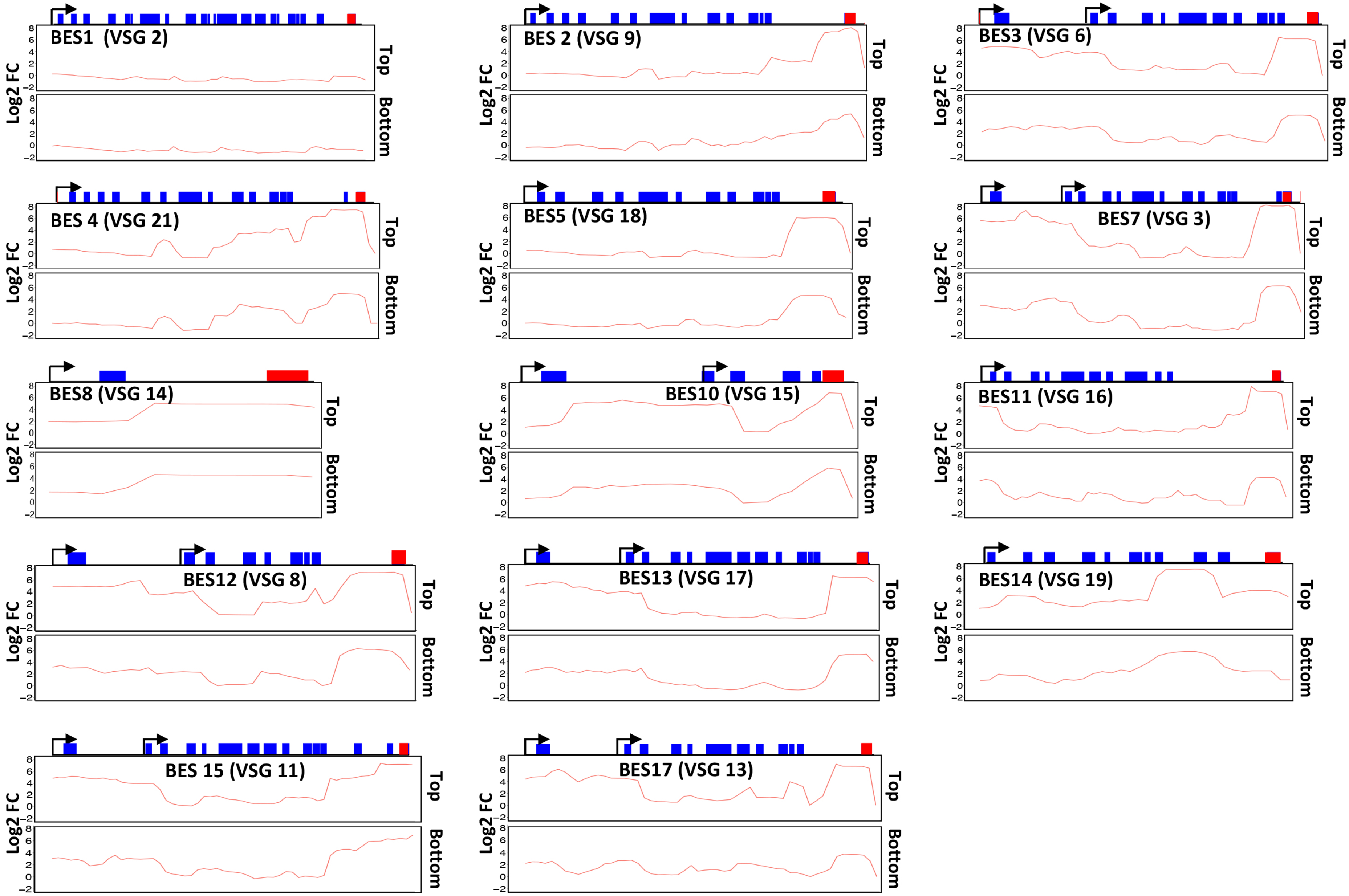
BES1 (VSG 2)
BES 2 (VSG 9)
BES3 (VSG 6)
BES 4 (VSG 21)
BES5 (VSG 18)
BES7 (VSG 3)
BES8 (VSG 14)
BES10 (VSG 15)
BES11 (VSG 16)
BES12 (VSG 8)
BES13 (VSG 17)
BES14 (VSG 19)
BES 15 (VSG 11)
BES17 (VSG 13)
Top
Bottom
Log2 FC
8
6
4
2
0
−2

**Figure S9**

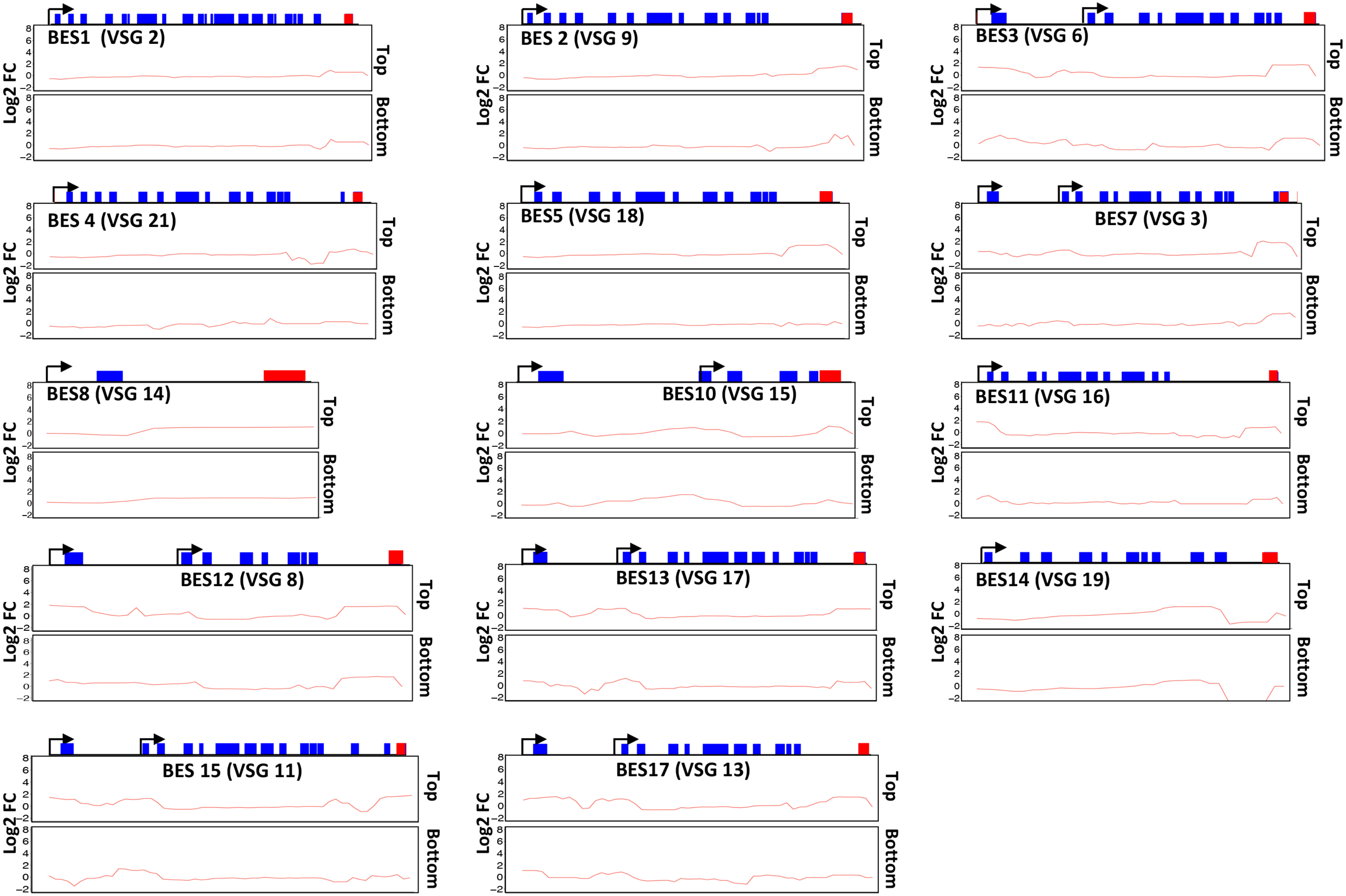
BES1 (VSG 2)
BES 2 (VSG 9)
BES3 (VSG 6)
BES 4 (VSG 21)
BES5 (VSG 18)
BES7 (VSG 3)
BES8 (VSG 14)
BES10 (VSG 15)
BES11 (VSG 16)
BES12 (VSG 8)
BES13 (VSG 17)
BES14 (VSG 19)
BES 15 (VSG 11)
BES17 (VSG 13)
Log2 FC
Top
Bottom
8
6
4
2
0
−2

**Figure S10**

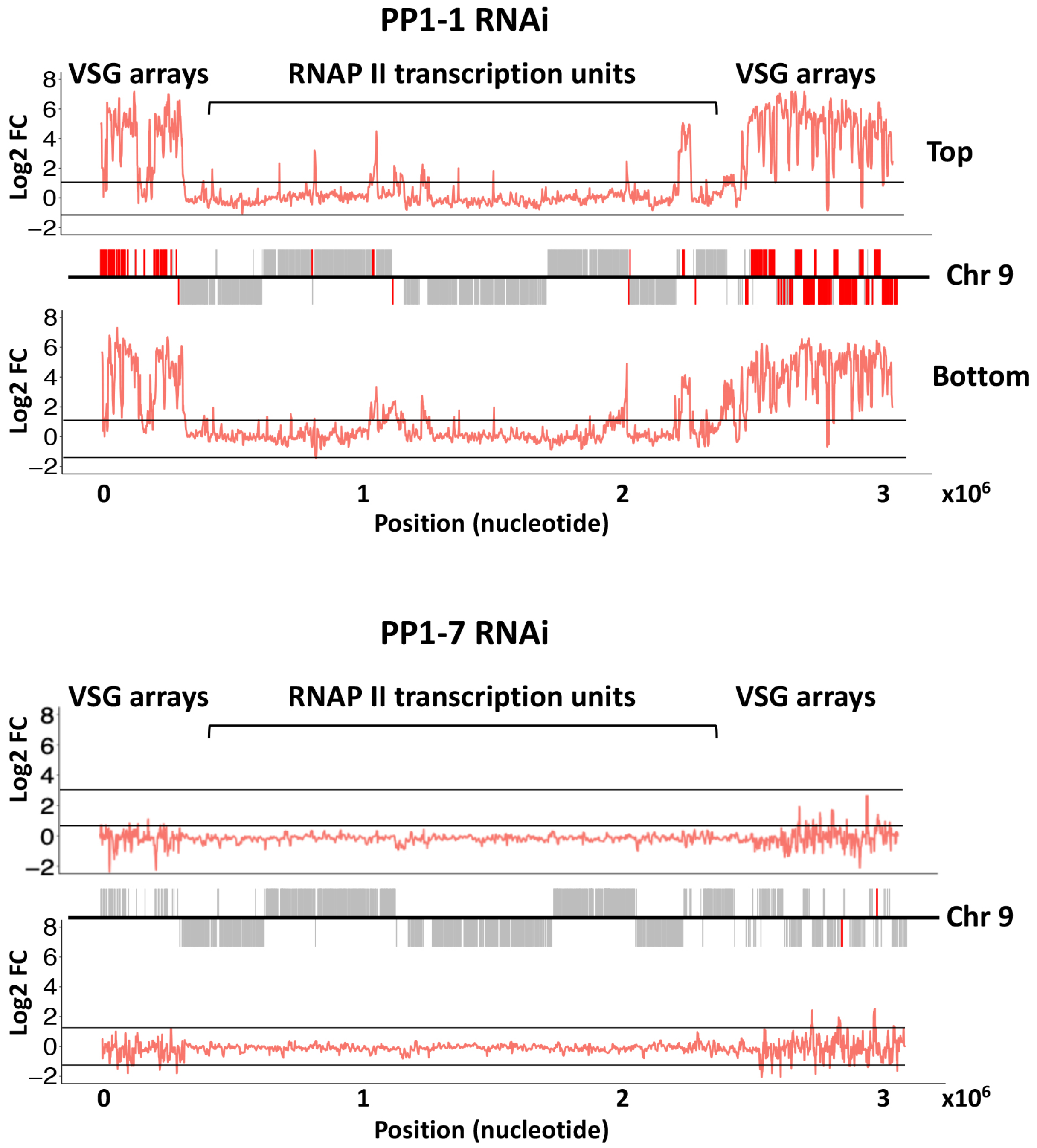
PP1-1 RNAi
VSG arrays
RNAP II transcription units
VSG arrays
Log2 FC
8
6
4
2
0
−2
Top
Chr 9
Bottom
0
1
2
3
x10^6
Position (nucleotide)
PP1-7 RNAi
VSG arrays
RNAP II transcription units
VSG arrays
Log2 FC
Chr 9
Position (nucleotide)

Figure S11

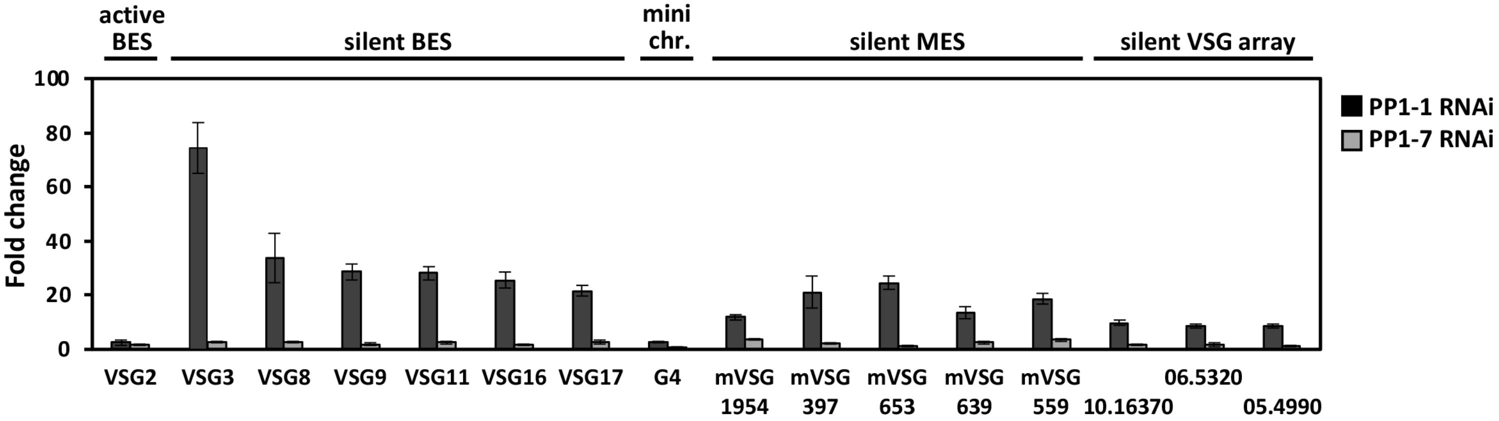
active BES
silent BES
mini chr.
silent MES
silent VSG array
100
80
60
40
20
0
Fold change
PP1-1 RNAi
PP1-7 RNAi
VSG2
VSG3
VSG8
VSG9
VSG11
VSG16
VSG17
G4
mVSG 1954
mVSG 397
mVSG 653
mVSG 639
mVSG 559
10.16370
06.5320
05.4990

**Figure S12**

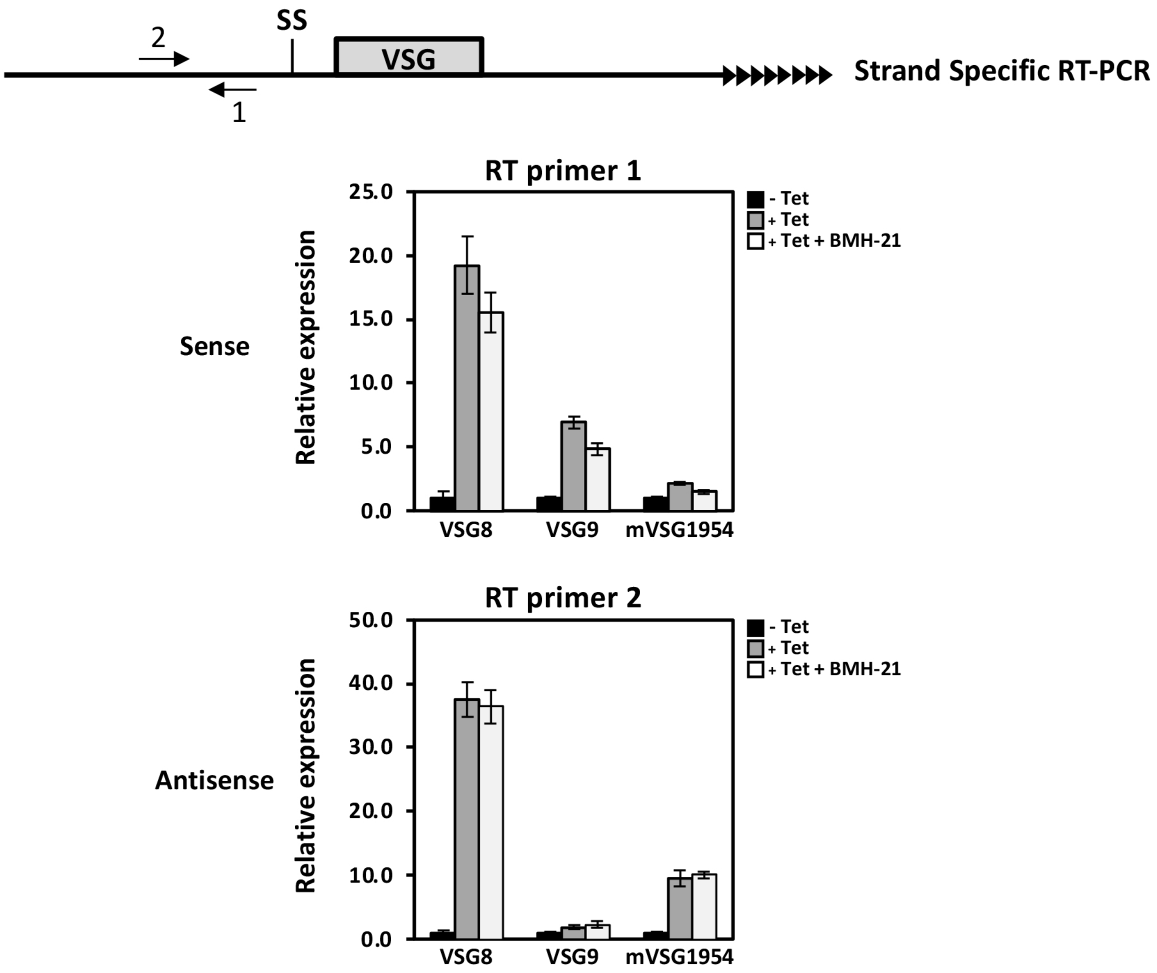
SS
2
VSG
1
Strand Specific RT-PCR
Sense
RT primer 1
Relative expression
25.0
20.0
15.0
10.0
5.0
0.0
VSG8
VSG9
mVSG1954
- Tet
+ Tet
+ Tet + BMH-21
Antisense
RT primer 2
Relative expression
50.0
40.0
30.0
20.0
10.0
0.0
VSG8
VSG9
mVSG1954
- Tet
+ Tet
+ Tet + BMH-21

**Figure S13**

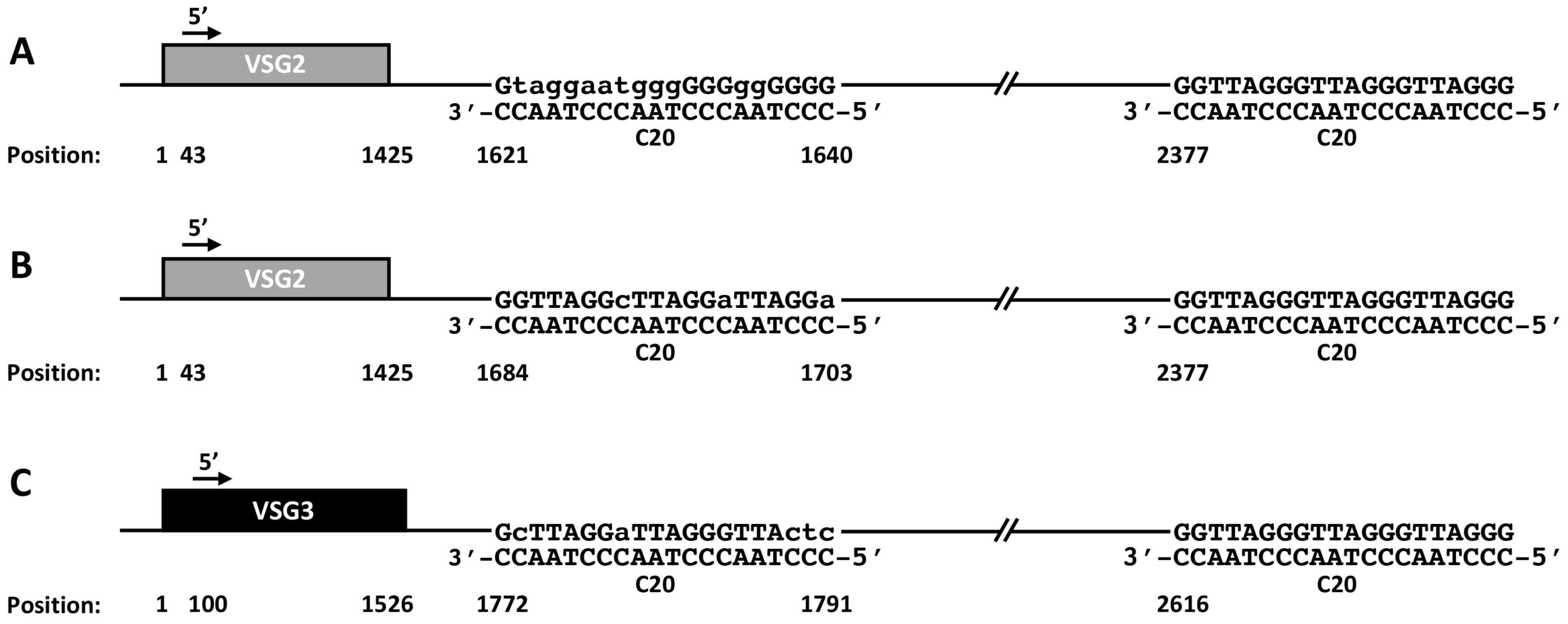
A
5′
VSG2
Gtaggaatgggg GGGggGGGG
3′-CCAATCCCAATCCCAATCCC-5′
C20
GGTTAGGGTTAGGGTTAGGG
3′-CCAATCCCAATCCCAATCCC-5′
C20
Position: 1 43 1425 1621 1640 2377
B
5′
VSG2
GGTTAGGcTTAGGaTTAGGa
3′-CCAATCCCAATCCCAATCCC-5′
C20
GGTTAGGGTTAGGGTTAGGG
3′-CCAATCCCAATCCCAATCCC-5′
C20
Position: 1 43 1425 1684 1703 2377
C
5′
VSG3
GcTTAGGaTTAGGGTTActc
3′-CCAATCCCAATCCCAATCCC-5′
C20
GGTTAGGGTTAGGGTTAGGG
3′-CCAATCCCAATCCCAATCCC-5′
C20
Position: 1 100 1526 1772 1791 2616

**Figure S14**

**A**

| | RVxF … $\Phi_R$ $\Phi\Phi$ Phe |
|---|---|
| LtPNUTS | $^{91}$APSRKRVCWADEGHTDVSRG.LVKHVTNFYMPNT.SR$^{125}$ |
| hPNUTS | $^{393}$RKRKKTVTWPEEGK........LREYF.YFELDET.ER$^{420}$ |
| TbPNUTS | $^{135}$ERRGKRISWHDDENGGVESDSLSSSSLRSGAKRRIDNMF$^{173}$ |

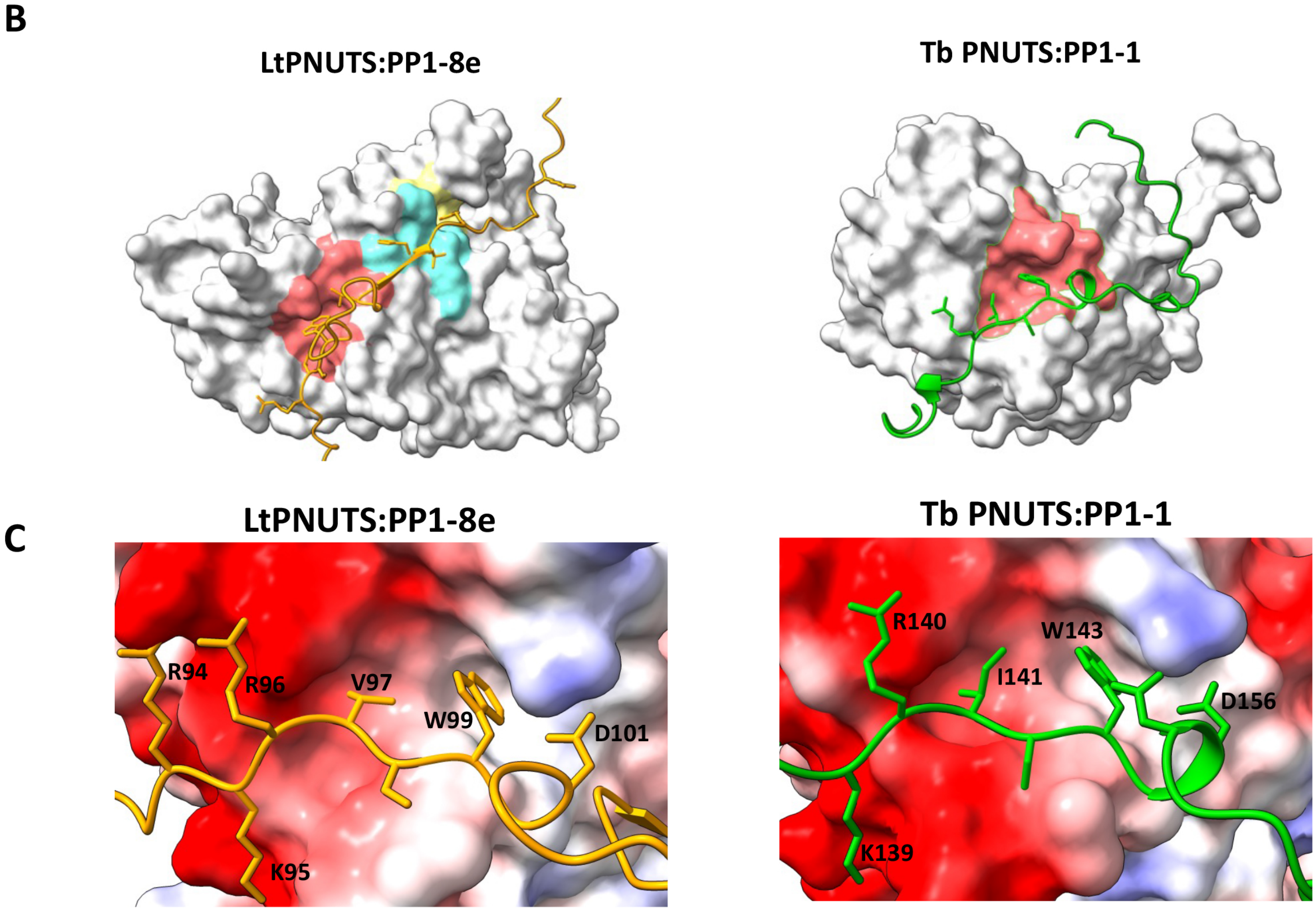


**Figure S15**

A

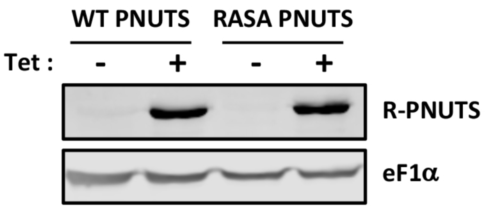


B

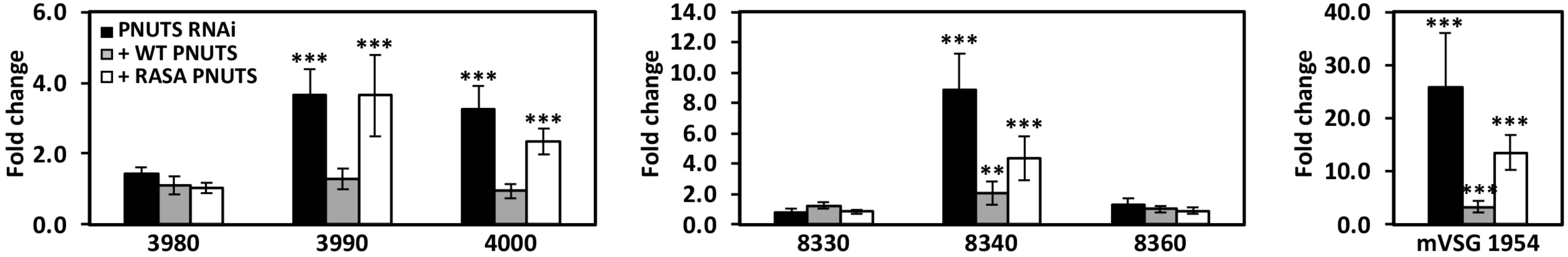


Figure S16

**Figure S1 Phylogenic analysis of PP1 phosphatase in *L. major* and *T. brucei*.** (A) The phylogenic tree was obtained as previously described (53). The five clades of PP1 (A-E) are indicated and T. brucei PP1 isoforms are in black and *L. major* isoforms in red. Black boxes indicate the LmPP1-8e characterized as a component of the PJW/PP1 complex involved in Pol II termination and shown to dephosphorylate Pol II (53) and two TbPP1 isoforms (PP1-1 and PP1-7) that localize to the nucleus (77). The PP1 protein from Saccharomyces cerevisiae (Glc7p) and Human are included for comparison. PP1 sequences from *T. cruzi*, in grey, are also included for comparison. Isoforms are not labelled for the *T. cruzi* proteins due to the lack of functional and gene annotation data. See text for details. (B) Visualization of protein abundance determined by proteomic studies between *in vitro* cultivated T. brucei bloodstream and procyclic lifestages using the protein abundance visualization tool (76). The protein abundance rank positions, from least to most abundant according to iBAQ values (blue circles). The TbPP1 isoforms are indicated.

**Figure S2 Sequence alignment of *T. brucei* PP1 isoforms and human PP1α.** Identical (*), conservative (:) and similar (.) residues between hPP1 and TbPP1 isoforms are indicated. Cylinders and arrows representing α-helices and β-strands of the hPP1 catalytic domain based on protein structure (3E7a), respectively, are drown under the sequence. The predicted TbPP1 models from the AlphaFold protein database were overlaid to the human PP1 structure to determine the positions of the secondary structures. Conserved α-helices and β-sheets are highlighted in green and yellow, respectively. Residues forming a unique helix in TbPP1-2 and TbPP1-3 not found in hPP1 are italicized. The six manganese-coordinating residues are underlined in red in the hPP1 sequence. TbPP1-8 is truncated at the N- and C-terminus of catalytic domain and lacks the sixth manganese-coordinating residue that is essential for catalytic activity.

**Figure S3 Specificity of clade specific PP1 RNAi screen**
(A) In vitro growth of the indicated clade specific PP1 RNAi cell line with (+Tet) and without (-Tet) induction of RNAi. Growth data are the means (and SEM) of three experiments done in triplicate. (B) Western blot analysis of the PP1-1 RNAi. Depletion of PP1-1 was induced in the RNAi cell line with PP1-1 and the indicated PP1 isoform endogenously tagged with HA and Myc, respectively. Quantitation of PP1 protein after 48 hrs induction of RNAi is indicated below. eF1α provides a loading control.
(C and D) TbPP1-1 affects early termination of antisense transcription at TSSs. (C) Representative region of chromosome 10 illustrating bi-directional transcription at a TSS. Thick black arrows indicate the start site and direction of sense transcription of the divergent PTUs. Thin black arrow indicates bi-directional non-productive transcription that typically terminates early. Red arrow indicates termination defect of the 'antisense transcription' upon depletion of PNUTS, Wdr82 or JBP3, resulting in de-repression of the annotated 8340 gene on the bottom strand (52). (D) mRNA transcript changes in the indicated PP1 isoform RNAi by RT-qPCR. RT-qPCR analysis was performed for the genes numbered according to the

ORF map. Transcripts were normalized against Asf1 and fold changes are plotted as the average and standard deviation of two biological replicates analyzed in triplicate.

**Figure S4 Time course of termination defects in the PP1-1 RNAi.** (A) Schematic representation of a termination site on chromosome 5 and primer location for analysis of readthrough transcription defect by RT-PCR as described in Figure 1. (B) Schematic diagrams of telomeric VSG Bloodstream-form Expression Sites (BES) as described in Figure 3. VSG2 is expressed from the active expression site. VSG8 is in one of the silent expression sites. (C) *In vitro* growth of the PP1-1 RNAi cell line upon Tet induction of RNAi. Mean and range values are plotted from two biological replicates. (D) RT-PCR analysis of nascent RNA from (A) at 0, 8, 16, 24 and 45 hrs of the PP1-1 depletion. cDNA was synthesized from the indicated timepoints using random hexamers and PCR was performed using the primers to amplify fragment 1, as described in Figure 1. Asf1 represents a PTU internal Pol II transcribed single copy gene as a loading control. (E) Quantitative analysis of termination defects during the time course of RNAi induction. Top (TTS1): Quantitative analysis of the changes in nascent RNA from (D), using ImageJ software from two biological replicates. Mean and range values are plotted. T=0 is arbitrarily set as 1. Middle and bottom: RT-qPCR analysis of mRNA transcript changes was performed for the gene indicated in (A; 3990) and a VSG gene from a silent BES (B; VSG 8). Transcripts were normalized against Asf1 and fold changes (+/- Tetracycline) are plotted as the average and standard deviation of two biological replicates analyzed in triplicate. T=0 is arbitrarily set as 1.

**Figure S5 PP1-1 can fully rescue the termination defect in the PP1-1 RNAi.** (A) Schematic representation of a termination site on chromosome 5 and primer location for analysis of readthrough transcription defect by RT-PCR as described in Figure 1. (B) Right; Induction of PP1-1 depletion and expression of the indicated recoded PP1 isoforms (PP1-1, PP1-7 or PP1-6). RT-PCR analysis of PP1 expression and readthrough RNA transcription defect (1) was performed for the indicated cell lines before and after 24 hr induction with Tetracycline (Tet). Asf1 mRNA was assayed as a loading control. Left; identical analysis was performed for WT *T. brucei* (SMC) cells transfected with the pLew100 construct allowing over-expression of the indicated PP1 isoform.

**Figure S6 PP1-1 can rescue the growth defect in the PP1-1 RNAi.** (A) Left; In vitro growth of the PP1-1 RNAi cell line with (+Tet) and without (-Tet) induction of RNAi. Right; growth of the PP1-1 RNAi cell line that also allow Tet inducible expression of the indicated RNAi resistant PP1-1 isoform. Growth data are the means (and SEM) of three experiments done in triplicate. (B) Growth of WT (SMC) *T. brucei* cells over-expressing the indicated PP1-1 isoform.

**Figure S7 Effect of PP1-1 KD on transcription termination.**

(A) Mean top strand coverage at each nucleotide position in the 10 kb surrounding the transcription termination site (TTS) at 39 cSSRs for the PP1-1 and PP1-7 RNAi cell line as described in Figure 2A. The

schematic represents the protein-coding genes associated with each strand at an "average" convergent TTS and arrows represent the direction of transcription. Plots are orientated that transcription proceeds from the left and terminates at "0", with the top strand being the coding strand on the left side of the TTS. Panels on the left depict reads mapping to the top strand, and panels on the right depict reads mapping to the bottom strand. (B) Box-and-whisker plots showing the median top and bottom strand coverage in the 5-kb region downstream of all 39 cSSR TTS for the PP1-1 and PP1-7 RNAi cell lines. Multiple comparisons were conducted by Wilcoxon test. P values are presented on top of each compared group.

**Figure S8 Quantitation of read through at cSSRs by RNA-seq before and after TbPP1-1 and TbPP1-7 depletion.** For 60 cSSRs (located within the chromosome core and are flanked by PTU containing at least 3 ORFs) in the *T. brucei* genome, total RNA-seq reads (RPM) that map to the cSSR were totaled for both top and bottom strand combined. Normalized reads per million estimates were derived for cSSRs as the average across replicates per sample. After the normalization, we averaged the normalized read coverage across all nucleotides within each cSSR region defined as a window that begins with end of each gene in the converging PTUs. Differences between + and – Tet were measured by Wilcoxon test based on the averaged-normalized coverage values. The results are shown for the indicated RNAi cell line grown in the absence and presence of tetracycline for 24 hrs.

**Figure S9 TbPP1-1 inhibits transcription of VSG BES.** RNA-seq reads from the PP1-1 RNAi were aligned to the *T. brucei* 427 BES sequences (14 BESs). Fold changes (Log2 FC) comparing plus and minus Tetracycline were plotted over each BES as described in Figure 3D.

**Figure S10 TbPP1-7 has no effect on transcription of VSG BES.** RNA-seq reads from the PP1-7 RNAi were aligned to the *T. brucei* 427 BES sequences and fold changes were plotted as in Figure S9.

**Figure S11 Transcriptome analysis of chromosome 9 after PP1-1 and PP1-7 knockdown.** Transcription was measured by stranded RNA-seq. Fold changes comparing transcription levels between – and + tetracycline induction of PP1-1 and PP1-7 RNAi for 48 hrs were calculated in 5000bp windows (1000bp step) and plotted over the length of chromosome 9. Forward (top strand) and reverse reads (bottom strand) were analyzed separately and plotted above and below the chromosome diagram, respectively. The transcriptome shows that ablation of PP1-1 leads to accumulation of transcripts at silent regions of the genome; PTU borders at the chromosome core and subtelomeric regions. Genes that were upregulated >3-fold upon ablation of the indicated factor are highlighted in red in the chromosome diagram.

**Figure S12 Ablation of TbPP1-1 results in de-repression of silent MES and BES VSGs.** qRT-PCR analysis of VSGs from subtelomeric arrays after 48 hrs of PP1-1 knockdown. Also shown is VSG RNA

from a minichromosome (VSG G4). Error bars indicate standard deviation from two biological replicates, measured in triplicate.

**Figure S13 Sense and antisense transcription of VSG BES following PP1-1 depletion is not affected by the Pol I inhibitor BMH-21.** Top, schematic representation of telomeric ends of VSG ES. The sketch is not in scale. Arrowheads represent telomeric repeats. SS; splice leader attachment site for the VSG gene. Arrows indicate primers used for ssRT-PCR analysis, where (for example) primer 1 used in the RT reaction, and both primers for the subsequent PCR reaction, allow analysis of nascent sense RNA upstream of the indicated silent VSG gene. In contrast, use of primer 2 in the RT reaction would allow analysis of anti-sense RNA. Below, pol II transcribed products within silent VSG ESs, upstream of the VSG gene, are insensitive to BMH-21. Levels of the indicated precursor transcript (sense and antisense) were measured in TbPP1-1 RNAi cells before and after induction of RNAi with and without BMH-21 treatment for 15 min. Error bars indicate standard deviation from at two biological replicates, measured in triplicate.

**Figure S14 Mis-priming by the TelC20 oligonucleotide.** (A) Diagram of the telomeric region of the active BES (VSG2). The 5' VSG primer is indicated by the arrow and the telomere primer C20 used in the RT and PCR reactions in Figures 6 and 7 is shown hybridized to the telomere (RNA) sequence. The sequence represents RNA sequence from transcription of the active ES. Position; represents bp relative to the 5' end of the VSG ORF. Hybridization of the C20 to the G-rich RNA from the telomere is shown on the right. Hybridization of C20 downstream of the VSG represents mis-priming by the oligo as indicated by DNA sequencing of the RT-PCR amplicon in Figure 7B. (B) as in (A), but potential mis-priming by C20 oligo indicated based on Blast analysis. (C) as above, but represents the silent BES VSG3 and potential mis-priming ability of C20 based on Blast analysis.

**Figure S15 Predicted TbPNUTS:PP1-1 interacting motifs.** (A) Structure-based sequence alignment of the PP1-interacting motifs of LtPNUTS, hPNUTS, and TbPNUTS, with PP1 interacting residues indicated. Residues underlined preceding the RVxF motif are basic residues shown to be important for PP1 binding in the hPP1-PNUTS and LtPP1-PNUTS complex. Potential $\Phi_R$, ΦΦ and Phe motifs in TbPNUTS are highlighted even though they are not supported by the AlphaFold PNUTS:PP1 complex (below). (B) Predicted structure of the LtPNUTS:PP1-8e complex (54) and TbPNUTS-PP1-1 complex. Left; LtPNUTS is shown as orange ribbon with key interacting residues shown as sticks and LtPP1-8e is shown as a grey surface. LtPNUTS residues $^{96}$RVCW$^{99}$ bind to the RVxF binding pocket in PP1 (red), LtPNUTS residues $^{114}$HV$^{115}$ bind to the PP1 ΦΦ binding pocket (cyan), and LtPNUTS residues F118 binds to the Phe binding pocket (yellow). Right; TbPNUTS is shown as green ribbon with key interacting residues $^{140}$RISW$^{143}$ bind to the RVxF binding pocket. (C) An electrostatic surface potential representation (positive, blue; negative, red) of the RVxF binding pocket in the LtPNUTS-PP1-8e complex (Left) and TbPNUTS-PP1-1 complex

(Right). All PNUTS residues highlighted, and corresponding PP1 binding pocket residues, have been demonstrated as essential for LtPNUTS:PP1-8e complex formation (54).

**Figure S16 TbPNUTS RVxF motif is essential for PNUTS function in Pol II transcription termination.** An additional TbPNUTS RNAi clonal cell line was analyzed for ability of WT PNUTS or RVXF mutant PNUTS (RASA) re-expression to rescue Pol II termination defects as described in Figure 9. (A) Western blot analysis of RNAi resistant recoded (WT and RASA mutant) PNUTS-PTP expression (R-PNUTS). Probing the same blot for eF1$\alpha$ provides a loading control. (B) qPCR analysis of Pol II termination defects as in Figure 8D. P values were calculated using Student's t test. **, $p$ value <0.01; ***, $p$ value < 0.001.
