# Supplementary material for: Protein phosphatase PP1 regulation of RNA polymerase II transcription termination and allelic exclusion of VSG genes in trypanosomes
Source: Nucleic Acids Res. 2024 May 23;52(12):6866–85. doi: 10.1093/nar/gkae392 (PMC11229358; doi:10.1093/nar/gkae392)
Supplement: gkae392_Supplemental_Files [file gkae392_supplemental_files.zip › Supporting Information Table legends.docx]

Supporting Information Table S1. *T. brucei* gene expression changes after RNAi depletion of PP1-1. The genes showing the largest (>2log fold change) average (between the three replicates) significant (*p*-value_adj_<0.001) increase in RNA abundance, as determined by RNA-seq analysis, are shown. Gene descriptions (annotation and accession number), logfold upregulation and *p*-value adj are also listed. Level of PP1 transcripts for all isoforms is shown at the bottom.

Supporting Information Table S2. *T. brucei* gene expression changes after depletion of PP1-7 as in Table S1.

Supporting Information Table S3. High-throughput sequencing information. Information about all sequencing data generated in this study is listed.

Supporting Information Table S4. Oligos used in these studies.
